# Hospital-acquired bloodstream infections in neurological and neurosurgical units in Hungary, 2005-2013

**DOI:** 10.1186/2047-2994-4-S1-P207

**Published:** 2015-06-16

**Authors:** R Szabó, A Kurcz

**Affiliations:** 1 Department of Hospital Epidemiology and Hygiene, National Center for Epidemiology, Budapest, Hungary

## Introduction

Hospital-acquired bloodstream infection (HABSI) is a serious complication of hospitalization with high associated morbidity and mortality, within and outside of the intensive care units. However, information related to HABSIs among neurological and neurosurgical patients are limited.

## Objectives

Our objective was to describe the epidemiological trends of HABSIs in this patient population in Hungary performed a nine-year descriptive epidemiological analysis.

## Methods

Patient data were recorded and submitted into the national surveillance database (National Nosocomial Surveillance system) by the local infection control practitioners from the reporting hospitals. Based on this database, descriptive statistics were performed using EpiData version 3.1 (http://www.epidata.dk).

## Results

The overall incidence rate was 0.4 HABSIs per 100 discharges in both ward types. HABSIs were considered primary in 66.8% among neurological patients and 58.9% among neurosurgical patients. For secondary HABSIs, the primary infection site was respiratory tract infection (19.5% and 17.8%) in both ward type. The most common pathogen was the *Staphylococcus aureus* (19.3%) in neurological wards and the coagulase-negative staphylococci (17.4%) in neurosurgical wards. The overall case fatality rate was 9.2%.

## Conclusion

During the study period, there was a significant increase in incidence trends and high case fatality rates in both ward types. Therefore, facilitating the implementation of existing national guidelines among healthcare and infection control practitioners is essential in order to reduce the incidence rates of HABSIs and to improve the quality of patient care.

## Disclosure of interest

None declared.

